# Week 48 efficacy of 900/100 mg daily of darunavir/ritonavir in treatment-experienced HIV-1 patients with virological success: DARDAR study

**DOI:** 10.1186/1758-2652-13-S4-P29

**Published:** 2010-11-08

**Authors:** L Schneider, A Houssaini, S Lambert, G Peytavin, R Agher, A Chermak, P Flandre, V Calvez, AG Marcelin, J Ghosn, C Katlama

**Affiliations:** 1 Pitié Salpêtrière Hospital, Infectious Diseases, Paris, France; 2 Université Pierre et Marie Curie-Paris 6, UMR S-943, INSERM, U943, Paris, France; 3 Pitié Salpêtrière Hospital, Virology Departement, Paris, France; 4 Bichat-Claude Bernard Hospital, Pharmacology Department, Paris, France; 5 Bicêtre Hospital, Medical Department, Le Kremlin-Bicêtre, Paris, France

## Background

Simplification of antitretroviral treatment (ARV) is particularly important. If darunavir/ritonavir (DRV/r) can be used at a 800/100mg once a day (q.d.) on patients with a wild-type virus, it is recommended at a 600/100mg twice a day (b.i.d.) on pre-treated patients. POWER study suggests the similarity of efficiency of the 800/100 mg q.d. and 600/100mg b.i.d. in patients with a minimal number of DRV resistance mutations.

## Objectives

Evaluate the capacity of DRV/r(900/100mg) q.d. to maintain viral load(VL) indetectability at W24, after switch from DRV/r 600/100mg bid, in virologically supressed pre-treated HIV-1 patients.

## Methodology

This observational study included 45 patients if they had a VL<50copies/ml and a steady treatment associating DRV/r 600/100 mg b.i.d. with INTI and/or INNTI. A genotypic test was perform on the plasmatic HIV-1 RNA, on the last detectable VL (>50cp/ml) before starting DRV, and in case of virological failure. The follow up is done at D0, W4/W12/W24/W36 and W48 including VL measure, CD4 cells count, residual plasmatic concentrations of darunavir and ritonavir. Virological failure was defined as two consecutives VL >50cp/ml at a minimal 15 days interval. The primary endpoint was the proportion of patients with a VL <50cp/ml at W24.

## Results

Between 02/2008 and 02/2009, 45 patients were included with an anterior ARV treatment of 13[1,20] years, with exposure to 3 ARV classes among 34( 75%) patients and a previous failure ≥ 2 PI for 20(44%) patients. CD4 cell count was 478/mm3 [317-560]; nadir CD4: 93/mm3 [39-165].They were treated by DRV/r b.i.d. since 10[3;44] months. DRV/r was associated with: 2 INTI (76%), 3 INTI (8%), 2 INTI/1INNTI( 5%). 93% plasmatic RNA genotypic tests were amplified. Five patients had ≥ 3 DRV impacting resistance mutations, six patients 2 mutations, and nine patients 1 mutation. The proportion of patients with VL<50cp/mL[IC95] at W24 was 93%[81-98] and 91%[78-97] at W48, in ITT and PP analysis.

Three virological failures were observed at W12 and one at W48. For 1 patient, 2 primary PI mutations (I50V and L33F) were observed.

## Conclusion

This study suggests that DRV/r can be used once a day even for patients with a previous IP failure. This approach is particularly important for the once daily use of combination INTI. It also enables a reduction of the ritonavir dose.

